# Retraction: An efficient one pot three-component synthesis of 2,4,6-triarylpyridines using triflimide as a metal-free catalyst under solvent-free conditions

**DOI:** 10.1039/d0ra90073g

**Published:** 2020-06-26

**Authors:** Laura Fisher

**Affiliations:** Royal Society of Chemistry Thomas Graham House, Science Park, Milton Road Cambridge UK advances-rsc@rsc.org

## Abstract

Retraction of ‘An efficient one pot three-component synthesis of 2,4,6-triarylpyridines using triflimide as a metal-free catalyst under solvent-free conditions’ by Hongshe Wang *et al.*, *RSC Adv.*, 2019, **9**, 5158–5163, DOI: 10.1039/C9RA00653B.

The Royal Society of Chemistry hereby wholly retracts this *RSC Advances* article. The NMR spectra for compounds 3a–3k and 3s–3u in the ESI have been reproduced without permission from a *Tetrahedron Letters paper* by Fei Ling *et al.*^1^ The authors stated that as their 2,4,6-triarylpyridines products are known, they only characterised three of their compounds using ^1^H NMR and ^13^C NMR, which were identical to those reported in the literature. Therefore, the authors do not have full characterisation data for their published paper.

Hongshe Wang opposes the retraction. Weixing Zhao, Juan Du, Fenyan Wei, Qi Chen and Xiaomei Wang were contacted but did not respond.

Signed: Laura Fisher, Executive Editor, *RSC Advances*

Date: 16^th^ June 2020

## Supplementary Material
